# Oral Microbiota, Its Equilibrium and Implications in the Pathophysiology of Human Diseases: A Systematic Review

**DOI:** 10.3390/biomedicines10081803

**Published:** 2022-07-27

**Authors:** Barbara Giordano-Kelhoffer, Cristina Lorca, Jaume March Llanes, Alberto Rábano, Teodoro del Ser, Aida Serra, Xavier Gallart-Palau

**Affiliations:** 1 Faculty of Dentistry, Universitat Internacional de Catalunya (UIC), 08017 Barcelona, Spain; bgiordano@uic.es; 2 Bioengineering Institute of Technology, Faculty of Health Sciences, Universitat Internacional de Catalunya (UIC), 08017 Barcelona, Spain; 3 Faculty of Health Sciences, Valencian International University, 46002 Valencia, Spain; 4 Biomedical Research Institute of Lleida Dr. Pifarré Foundation (IRB Lleida), Neuroscience Area, +Pec Proteomics Research Group (+PPRG), University Hospital Arnau de Vilanova (HUAV), 25198 Lleida, Spain; cristina.lorca@imdea.org; 5 IMDEA—Food Research Institute, +Pec Proteomics, Campus of International Excellence UAM + CSIC, Old Cantoblanco Hospital, 8 Crta. Canto Blanco, 28049 Madrid, Spain; 6 NeuroPGA Research Group—Psychology Department, University of Lleida (UdL), 25001 Lleida, Spain; jaume.march@udl.cat; 7 Alzheimer’s Centre Reina Sofia—CIEN Foundation, 28031 Madrid, Spain; arabano@fundacioncien.es (A.R.); tdelser@fundacioncien.es (T.d.S.); 8 Psychology Department, University of Lleida (UdL), 25001 Lleida, Spain

**Keywords:** oral microbiota, gut microbiota, caries, periodontal disease, dysbiosis, cardiovascular disease, neurodegeneration, Alzheimer’s disease

## Abstract

Imbalances of the oral microbiota and dysbiosis have traditionally been linked to the occurrence of teeth and oral diseases. However, recent findings indicate that this microbiota exerts relevant influence in systemic health. Dysbiosis of the oral microbiota is implicated in the apparition and progression of cardiovascular, neurodegenerative and other major human diseases. In fact, the oral microbiota are the second most diverse and largely populated microbiota of the human body and its relationships with systemic health, although widely explored, they still lack of proper integration. The purpose of this systematic review is thus to widely examine the implications of oral microbiota in oral, cardiovascular and neurodegenerative diseases to offer integrative and up-to-date interpretations. To achieve that aim, we identified a total of 121 studies curated in PUBMED from the time interval January 2003–April 2022, which after careful screening resulted in 79 studies included. The reviewed scientific literature provides plausible vias of implication of dysbiotic oral microbiota in systemic human diseases, and encourages further research to continue elucidating the highly relevant and still poorly understood implications of this niche microbiota in systemic health. PROSPERO Registration Number: CRD42022299692. This systematic review follows relevant PRISMA guidelines.

## 1. Introduction

Commensal microorganisms, within the human body, have traditionally been regarded as foes on their way to abolition [1]. However, further and more recent research has proven these initial considerations to be wrong and novel essential role(s) attributable to human microbiota, in health and disease conditions, are appearing [2,3,4]. Currently, it is well known that we as humans are holobionts from our birth; thus, we should be considered as complex organisms composed of multiple cells and genomes of eukaryotic and microbial origins [5,6]. These cells, in turn, structure the different organs and systems of the human body and, most importantly, contribute to their proper and homeostatic functioning [1,7]. Therefore, the human microbiota richness and variety is an inseparable part of a dynamic organism and its functions, and its alterations are known to involve dysbiosis [1,8].

Dysbiosis in different organismic systems has in turn been proposed as a core contributor to the apparition of several diseases [9,10,11]. A clear example of the effects of dysbiotic microbiota in the human organism has been revealed in the gut [12]. This organ system contains the most extensive number of microbial cells, with more than 1000 different species [13]. Alterations in the compositions of the gut microbiota, as those caused by unhealthy diet and genetic predisposition [14,15], drive to microenvironment disequilibrium and apparition of disorders and diseases such as obesity, colorectal cancer, inflammatory bowel disease [3], heart failure, diabetes and neurodegeneration [14,16,17]. The gut microbiome is also known to influence the central nervous system (CNS), contributing to its proper functioning [18,19,20], and to the integrity of the blood–brain barrier (BBB) [21,22]. Induction of brain neuroinflammation as a consequence of alterations in gut bacteria populations has been reported and integrity of the BBB implicated; however, the mechanisms by which these pathological interactions may take place remain poorly defined [17]. Some studies indicate that bacteria metabolites [18,22,23] or the enzymatic effects of matrix metalloproteinases of bacterial origin [22] may alter the integrity of the BBB and contribute to increase the permeability of the BBB promoting pathologic leakage of diverse molecules and chronic neuroinflammation [22].

Although several studies have linked dysbiosis with the apparition of major diseases as indicated, it is not yet well understood how the gut microbiome can affect these distant organs and systems. Translocation of bacterial products by the gut endothelial barrier, however, is being considered as a plausible hypothesis [24,25]. Moreover, communication between the gut and the brain has been proposed to partially occur mediated by the vagus nerve. This nerve bundle directly connects the digestive and nervous systems in the mammal body; thus, bacterial species and/or metabolites may use it for bi-directional communication [17,18].

## 2. Oral Microbiome and Systemic Diseases

Following the gut microbiome, the second most diverse and largely populated microbiome in the mammal body is in the oral cavity [26,27,28,29,30]. Although gut and brain interactions have been explored, the interactions between the oral microbiome and the brain still remain mostly unexplored. The interest in the oral cavity microbiome, however, has increased in recent times, considering the proximity of this microbiota to the brain. Research on the oral microbiota has revealed that the oral microbiome is highly dynamic and becomes influenced by different lifestyle aspects, such as diet, stress, tobacco consumption and systemic conditions, which actively modify its composition and characteristics of this microbiome by even misleading it to dysbiosis [31,32,33]. The interaction of diet and microbiome can be a relevant risk factor for multiple systemic diseases, such as oral diseases, obesity, cardiovascular and neurodegenerative diseases, among others [34], and dietary modifications can prevent some of the symptoms related to these pathologies, treating or alleviating them, and thus, it may result in improved overall health [8].

### 2.1. The Microbiota of the Oral Cavity

**Figure 1:**
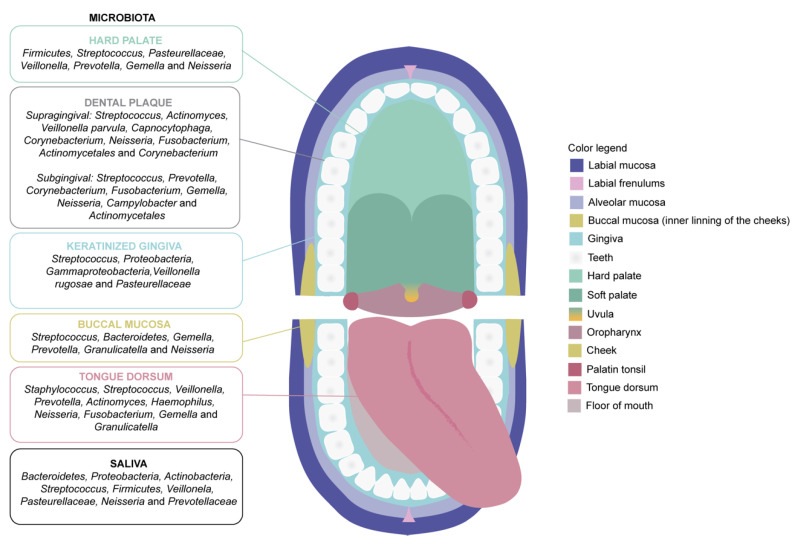
Illustrative diagram depicting the diverse microbiota populations that form the niche oral microbiota throughout the different oral cavity regions.

The microbiome compositions are distinct in each of the areas of the digestive tract, oral cavity, esophagus, stomach, intestine and colon, and become influenced by the ecosystem of each digestive organ [35,36]. Among the components that form these ecosystems, the oral cavity contains the exclusive presence of hard and soft tissues (e.g., teeth and oral mucosa, respectively) [26,37]. The oral mucosa is composed of the lining mucosa (floor of the mouth, buccal region, labial region and soft palate), masticatory mucosa (gingival region and hard palate) and the specialized mucosa (back of the tongue) (Figure 1). The mucosa of the lining is covered by a non-keratinized epithelium, while the masticatory mucosa is covered by a keratinized epithelium. The surface of the specialized mucosa presents a complex structure, containing different types of papillae [38,39]. These differences may be behind the diversity in terms of microbiota niches that can be found within the oral cavity in normal conditions. Of note, the bacterial composition of dental surface plaque differs between the supragingival and subgingival region (Figure 1). In addition, the bacterial composition of saliva includes bacteria separated from various niches in the oral cavity and it is similar to that of the lining of the tongue [40], as detailed in Figure 1. For that reason, the importance of further defining the oral microbiota by embracing the complexity of the oral cavity should be highlighted, which in turn may contribute to improve our understanding on the basis of any potential occurring associations between the oral microbiota and general health [35].

The oral microbiota is composed of various microorganisms, such as bacteria, fungi and archaea [26,40]. This symbiotic community, in homeostatic equilibrium, is supposed to contribute to a healthy oral environment that has a systemic impact, as previously reported [11,33,41]. However, alteration of the homeostatic equilibrium of the oral microbiota, such as in dysbiosis, has been implicated in the apparition and progression of several diseases of the oral cavity and has been suggested to have a severe and harmful imprint on overall health [32]. Interestingly, oral cavity-associated microbes have been found in many distant organ sites, such as small intestines, heart, lungs, placenta and brain [33,42]. Oral microbiota, thus, show an enormous interactive communication and influence over local and systemic responses, and play essential role(s) in host nutrition, immunity, metabolism and diseases, as detailed in Table 1. Although establishing an oral microbiota profile in oral and general health conditions is preferential and several efforts have been already made towards that aim, recent studies already focus on a step further, which implies the analysis and characterization of the oral microbiota in systemic disease conditions [10,43,44].

The oral bacterial ecosystem comprises more than 700 microbial species that form a vast network of interactions [35,39,45,46,47]. In the state of oral health, there is homeostasis in the bacterial ecosystem and it provides a dynamic balance with mutual benefits for the host and microorganisms [45]. The presence of the commensal microbiota in the oral cavity prevents colonization of the region by pathogenic microbes. For example, secretion of bacteriocins by the genus *Streptococcus* prevents the colonization of Gram-negative bacteria, conferring a fundamental healthy advantage to the host [48].

### 2.2. Diseases Related to the Oral Microbiota

#### 2.2.1. Oral Diseases

##### Dental Caries and Tooth Decay

The number of studies suggesting that the composition of the oral microbiota and the metabolic potential of saliva and dental plaque vary significantly in health compared to disease conditions has tremendously increased in the last years [49]. A clear example of that research is tooth decay, which refers to an irreversible demineralization of the tooth hard tissues: enamel and dentin. Tooth decay is due to the production of organic acids by the bacteria that form the bacterial plaque. This process occurs through anaerobic metabolism of sugars ingested in the diet, especially following consumption of sucrose [50,51,52]. This fact accounts on the importance of the dietary patterns and the effects that these directly exert on the equilibrium of the oral microbiota. In a related vein, caries has been identified as the most prevalent non-communicable pathology at a global scale [53]. It is known that about 2.3 billion adults have cavities that affect their permanent teeth, and about 530 million children present cavities in their temporary teeth [54,55,56]. The current established mechanisms available to counteract the apparition and progression of that pathology are based on improved oral hygiene and fluoride administration [57]. However, in many of these cases, imbalances between the dynamics of enamel demineralization–remineralization and other protective factors have been identified [57]. Further research and appropriate dietary recommendations [58], thus, need to be implemented to manage the expanding global burden of tooth decay.

##### Periodontal Disease

Periodontal disease or periodontitis is a chronic immunoinflammatory pathology also considered a bacterial disease with a multifactorial cause. The pathogens that cause periodontitis are mainly anaerobic Gram-negative bacteria [17,59]. The high immune response that the presence of these bacteria causes withing the oral gums leads to high production of pro-inflammatory cytokines, such as tumor necrosis factor-α (TNF-α), as a response from the host immune system [60,61]. Periodontitis is also associated with increased serum levels of C-reactive protein (CRP) and decreased anti-inflammatory markers such as interleukin-10 [61]. This results in a first stage of severe gingival inflammation, followed by an irreversible loss of the tooth supporting tissues (the periodontal ligament and alveolar bone) [62,63]. Aggravating factors for this pathology are poor oral hygiene, tobacco habits, stress and disease comorbidities, such as type 1 and type 2 diabetes mellitus, cardiovascular disease and osteoporosis [60,64]. The prevalence of periodontitis is globally high, affecting between 35 to 50% of the worldwide population, as recently informed by the World Health Organization [53,65,66].

#### 2.2.2. Systemic Diseases

Varying levels of tooth decay, gingivitis and periodontitis, as well as further comprehension achieved on the role(s) of bacteria in pathological conditions, reveals that oral microbiome possesses fascinating role(s) affecting human systemic health beyond the oral cavity [67,68]. In fact, it has been proposed that oral microbiome products, microorganisms and inflammatory molecules could reach distal organism systems and organs through two different ways, mainly by the bloodstream and the digestive tract [11,32]. Table 2 provides details of different oral microbiome-related disorders that influence the occurrence and progression of human systemic diseases [1,33,43,69,70,71,72].

##### Cardiovascular Diseases

Arteriosclerotic cardiovascular disease (CVD) is a chronic condition that can lead to life-threatening clinical pathologies, such as acute myocardial infarction or stroke. These pathologies are responsible for a growing global burden of deaths and figure as the second global cause of mortality worldwide following sepsis [64,76]. Although the most well-known risk factors for the condition are hypertension, hypercholesterolemia and smoking, it is likely that other concomitant risk factors that remain poorly understood [77] may also be implicated. Numerous epidemiological studies have implicated oral dysbiosis and infection, specifically periodontal disease, as a significant risk factor associated with CVD [78,79,80]; the prevalence of CVD in patients diagnosed with periodontitis is 25–50% higher than in healthy individuals, although the mechanisms of this significant association are unclear [70]. Periodontal disease caused by dysbiosis and extended oral infection places a vast population of harmful bacterial strains and their secretions, which might involve the presence of extracellular vesicles [81], in direct contact with disrupted capillary terminals, which in turn introduces these harmful agents into the circulatory system facilitating a global systemic diffusion [60,79,82]. Thus, this systemic exposure to oral bacteria and their products probably exerts a significant impact on the onset and progression of CVD through activation of pro-inflammatory processes [42,83]. Although inflammation is induced by bacteria in the biofilms that comprise the microbiome formed on the teeth [79] and by the tongue-coating microbiota [84], these may not be the unique culprits. A genetically dependent effect on the immune mechanism or a modified immune reaction to the presence of pathogenic bacteria in the circulatory system may also be involved [70,78], a potential hypothesis that deserves further research.

##### Neurodegenerative Diseases

Neurodegenerative diseases involve a progressive loss of neurons in the central nervous system (CNS), resulting in impairment of several cognitive and motor domains and the apparition of dementia and movement disorders. Major neurodegenerative diseases include Alzheimer’s Disease (AD), Parkinson’s Disease, Huntington’s Disease, Amyotrophic Lateral Sclerosis and Multiple Sclerosis, among others [85,86,87]. It should also be noted that vascular factors associated with cerebrovascular disease are also a major cause of cognitive decline in elders and relevant risk factors for neurodegeneration [88,89,90,91]. The implications that oral microbiota homeostasis and dysbiosis may exert on the pathological mechanisms of these diseases, and more specifically in neuroinflammation, will be specifically addressed in this work through a systematic review.

## 3. Materials and Methods

### 3.1. Study Design and Protocol Registration

A systematic review protocol was designed and registered in PROSPERO (URL: https://www.crd.york.ac.uk/prospero/ (accessed on 30 May 2022), York, UK) with the identifier reference: CRD42022299692, to be carried out in PubMed. Only studies performed on humans and animals as well as review articles/metanalyses evaluating these populations were included.

#### 3.1.1. Inclusion and Exclusion Criteria

Studies meeting the following criteria were included for systematic revision: (a) articles and systematic reviews published in English; (b) articles and systematic reviews published in peer reviewed journals; (c) articles and systematic reviews published between January 2003 and April 2022; (d) Participants in the study: humans of all ages and mammalian animal models.

The exclusion criteria were established as follows: (a) in vitro and ex-vivo studies; (b) Grey literature considered as unpublished results, articles published in non-peer-reviewed sources, and studies published in indexed journals (Journal Citation Reports) with an impact factor lower than 1.7; (c) scientific literature falling out of the scope of the revision after consensual exclusion recommendation by two independent reviewer authors; (d) scientific literature published non-open access.

#### 3.1.2. Search Strategy, Risk of Bias and Limitations

Searches were performed by aleatory combination of the following keywords: “diet”, “oral microbiome”, “oral health”, “oral disease”, “nutrition”, “oral dysbiosis”, “cardiovascular disease”, “Alzheimer disease” and “neurodegenerative disease”; the Boolean operator AND was used to establish combinations of keywords. Further assessment of the articles and review articles retrieved by the previous referred parameters was performed by careful screening of the title, abstract and keywords to confirm their relevance and suitability. When the title and abstract did not provide enough details to include/exclude the study, the full text was assessed.

The exclusive inclusion of scientific literature published in open access may create a risk of bias resulting from the exclusion of relevant scientific findings not published through this option. However, we strongly believe that open access is an option of scientific dissemination emphasized and prioritized by funders and authors worldwide, which has been exclusively considered here, as it allows easier management of the literature reviewed and avoids specific bias resulting from non-institutional allowed access to certain resources published under subscription. Finally, the authors declare that misleading interpretation of the reviewed scientific literature and inappropriate extrapolation of findings, as previously reported by Ducker et al. [92], have been specifically addressed and, to the best of our ability, avoided.

## 4. Results and Discussion

**Figure 2:**
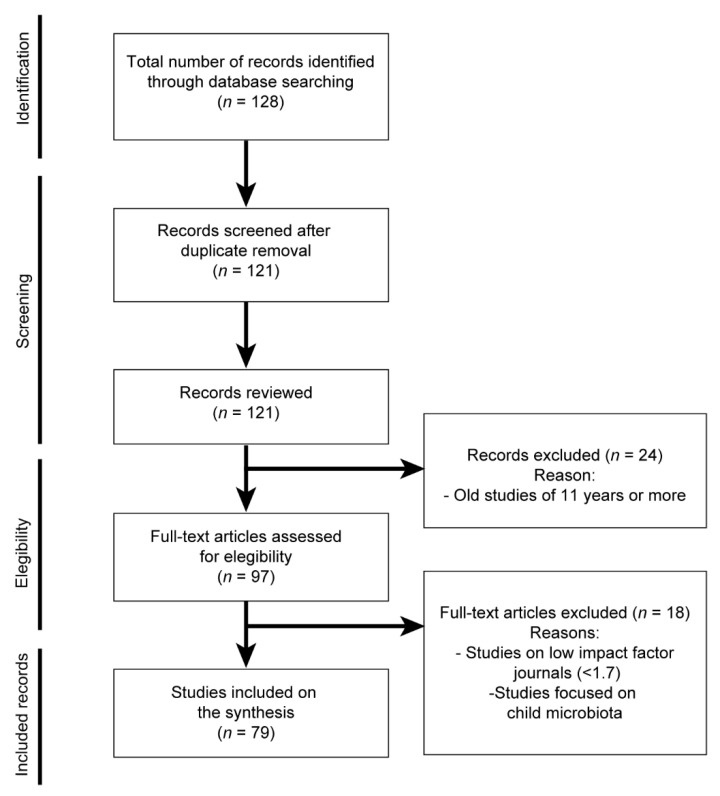
PRISMA flow diagram indicating the steps followed during the scientific literature review process of this work.

A total of 128 studies were initially obtained and pooled for further screening based on implementation of the above indicated inclusion and exclusion criteria as detailed in Figure 2. From these, 7 studies were removed during duplicate filtration, obtaining a remanent of 121 studies, from which 97 studies were published in the last 19 years. Additionally, 43 additionally studies were added manually. Thus, after careful screening, a total of 139 studies successfully passed the inclusion criteria and have been reviewed in this work, as detailed in Figure 2.

### 4.1. Oral Microbiota, Oral Diseases and Cancer

The oral cavity and its microbiome colonies are openly exposed to the external environment, which differentiates this specific region from the rest of digestive tract regions, and which poses a challenge to the microbiota of preventing external pathogenic colonization [27,31,44]. Further than preventing external colonization, imbalanced oral microbiota based on the most recent studies has been directly implicated in the apparition of multiple oral pathologies [4,31,33,46]. Tooth decay and periodontitis are the most common and costliest chronic oral pathologies, while oral cancer has also been linked to the presence of buccal dysbiosis [29,44,93,94]. In line with these findings, several studies provide association between periodontal disease and increased risk of cancer affecting distant organs [95,96]. Furthermore, specific oral microbiome dysbiosis patterns have been related to several types of cancer. Of note, augmented colonization of the oral microbiome by *T. forsythia* and *P. gingivalis* have been implicated in esophageal cancer [97], *P. gingivalis* and *A. actinomycetemcomitans* have been linked to pancreatic cancer [98] and genera *Fusobacterium* and *Porphyromonas* have been implicated in colorectal cancer [99], among others. Schwabe et al. proposed that the synergistic effects that eukaryotic and human cells take in human metabolism through the oral cavity, once imbalanced, could result in the apparition of carcinogenesis [100]. Abusleme and colleagues [101] investigated oral subgingival species and their association with periodontitis in a cohort of 22 subjects with chronic periodontitis and 10 periodontally healthy control subjects. Subgingival communities in this study were characterized using the 16S rRNA gene and quantitative PCR [101]. They observed that the increase in inflammation, linked to augmented bleeding, was not associated with a different microbiome, but corresponded to a higher community biomass in the bleeding areas. Moreover, oral microbiota showed clear differences in diversity and biomass; periodontic microbiota had an increase in the genus *Tannerella forsythia*, *Treponema denticola* and *P. gingivalis*, among others, compared to healthy microbiota.

Similarly, Yamashita and colleagues [35] studied the saliva microbiome of a Japanese population of 2343 adults using 16S rRNA gene and quantitative PCR. In this vast study, the authors found that high bacterial diversity in the saliva microbiome was significantly associated with poor oral hygiene, including presence of tooth decay and periodontitis. In addition, *Streptococcus mutans* (*S. mutans*) and *P. gingivalis* were identified in this study in higher frequency in subjects suffering from caries and periodontitis, which has also been previously demonstrated in vivo [41]. Yamashita and colleagues [35] also reported an association between the relative abundance of the predominant bacteria in saliva with conditions related to oral hygiene. Salivary microbiota organisms were present in the majority of individuals, including *Streptococcus*, *Rothia*, *Neisseria*, *Actinomyces*, *Prevotella*, *Granulicatella*, *Porphyromonas* and *Haemophilus*. Moreover, the information on the bacterial composition of the participants suggests that the predominant organisms encompass two groups of cohabiting bacteria, i.e., group I: *Prevotella histicola*, *Prevotella melaninogenica*, *Veillonella atypica*, *Veillonella parvula*, *Streptococcus salivarius* and *Streptococcus parasanguinis* and group II: *Neisseria flavescens*, *Porphyromonas pasteri*, *Haemophilus parainfluenzae*, *Granulicatella adiacens* and *Gemella sanguinis.* Based on this classification, predominance of species from group I in oral microbiome was associated with worsened health, such as presence of caries, periodontal disease and poor oral hygiene [35].

For a long time, the main bacteria strain considered cariogenic due to the production of acids and demineralization of the dental structure was *S. mutans* [74,94,102]. Instead, in recent decades, a more complex composition of the bacterial community associated with caries in its different stages of evolution has been considered and now includes the presence of *Streptococcus sobrinus*, *S. salivarius*, *S. parasanguinis*, *Actinomyces* and *Lactobacillus spp.* at the onset of caries [37,55,103], and *Veillonella*, *Propionibacterium*, *Bifidobacterium* and *Atopobium* in more advanced stages [55,103,104]. On the contrary, periodontitis-associated species include, but are not limited to, *P. gingivalis*, *Tennerella forsythia* and *Treponema denticola*, while *S. mutans*, *Lactobacillus spp.*, *Bifidobacterium spp.* and *Atopobium spp.* are associated with tooth decay [48,94].

Although one of the main bacteria associated with tooth decay is *S. mutans*, the genus Lactobacillus has been proposed as a possible probiotic agent against *S. mutans* [105]. Zhang et al. evaluated the presence of the genus Lactobacillus in a type of pickles to know their inhibitory properties on *S. mutans* both in vitro and in vivo. In that study, *L. plantarum* K41 strain showed the greatest inhibitory effect against *S. mutans* and also in the formation of exopolysaccharides and biofilm in vitro. In addition, the authors observed a significant reduction in the severity and incidence of tooth decay when treating rats with *L. plantarum* K41 strain [105].

It has also been demonstrated that different diet components can prevent or promote the development of caries [45]. In general, fiber-rich foods are known to stimulate the flow of saliva, buffering pH and protecting teeth [106,107]. On the other hand, plant phosphates (to a greater extent phytates) are able to reinforce teeth remineralization [45,108]. Some plant components, such as flavonoids and other polyphenols in blueberries, help reduce the risk of tooth decay by reducing the adhesion of bacteria, inhibiting their growth, or by reducing the ability of bacteria to form biofilm [45]. There are also several studies on the relationship of vitamins and periodontal disease, both in animals and humans [109,110,111,112,113]. Among all the vitamins analyzed, those with antioxidant capacity and with effects on the immune system, as well as those involved in bone metabolism, seem useful for the prevention or improvement of periodontal periodontal disease, highlighting the action in oral health of the vitamins C and D [114].

In this line, Anderson et al. [55] investigated the modification of the composition of bacterial plaque during frequent carbohydrate consumption. Participants continued with their usual diet and suctioned 2 g of sucrose five times a day between meals for a period of 3 months. Samples of biofilms were collected from splint systems worn for 3 × 7 days with 7-day intervals. Changes in the microbiota, investigated using Illumina MiSeq amplicon sequencing (v1–v2 region), revealed that consumption of sucrose for three months significantly altered the oral microbiota. These alterations included a general decrease in the richness of bacteria, directly associated to the sucrose intake, and a significant proliferation of *Streptococcus gordonii*, *S. sanguinis* and *S. parasanguinis*. In addition, a proliferation of acidic and acidogenic species, which cause dental caries, and a decrease in Porphyromonas, associated with typical healthy oral microbiomes, were also detected [55].

### 4.2. Oral Microbiota and Cardiovascular Diseases

It has been suggested that some bacteria, such as the aforementioned *P. gingivalis*, may systemically increase the risk to suffer certain cardiovascular diseases, being involved in the metabolic syndromes and autoimmunity, by causing alterations in the amino acid metabolism and the host’s immune response [30,93]. The *Firmicutes/Bacteroidetes* ratio may be another of the multiple contributing factors to cardiovascular diseases development and progression, since it has been reported that the increased *Firmicutes/Bacteroidetes* ratio in the oral microbiota may indicate an upregulation in the systemic inflammatory response mediated by pro-inflammatory cytokines [28,48]. Epidemiological studies indicate that various types of bacterial infection (*Helicobacter pylori, Chlamydia pneumoniae, P. gingivalis*, *Fusobacterium nucleatum*, *Aggregatibacter actinomycetemcomitans* and *Prevotella intermedia*) and the presence of metabolites derived from these bacteria in serum, such as lipopolysaccharides (LPS), contributes to the development of arteriosclerosis [70,115,116]. In addition, another study states that inflammatory risk factors for myocardial infarction have a systemic profile similar to those of periodontitis, suggesting a common final pathway of atherogenesis related to systemic inflammation [117]. The oral microbiome modulate oral immunity, but also the gut microbiome [46]; it can induce dysbiosis in the gut microbiota, leading to the disruption of the intestinal barrier and systemic inflammation [46,48,84,107]. Moreover, inorganic nitrate, found in high concentrations in meat, vegetables such as beets, lettuce and spinach and drinking water [118,119], has been studied as a potential prebiotic for oral microbiota [120]. In a clinical study focused on the cardiovascular benefits of dietary nitrates, oral bacterial profiles were measured [121]. Two nitrate-reducing species, *Rothia mucilaginosa* and *Neisseria flavescens*, were significantly increased in 65 hypercholesterolemic subjects who randomly daily received 250 mL of nitrate-rich beetroot juice or placebo juice daily during 6 weeks [121]. In addition, the presence of these bacterial strains is also associated, as a concomitant factor, with teeth and periodontal disorders [122]. Likewise, another study analyzed the microbiota present on the surface of the tongue of subjects who followed a diet with beetroot juice enriched in inorganic nitrate for 10 days by sequencing the bacterial genes 16S rRNA [123]. The results of this study showed that high prevalence of oral bacteria of the genus *Prevotella* and *Veillonella* should be considered harmful, while a high amount of the genus *Rothia* and *Neisseria* were beneficial for the maintenance of nitric oxide homeostasis and associated rates of cardiovascular disease and improved blood pressure [123].

### 4.3. Oral Microbiota and AD Neurodegeneration

AD is the main contributor to dementia worldwide and the fifth leading cause of death in people over 65 years [124]. Recently, a hypothesis has emerged that resident bacterial populations contribute to the development and progression of AD by promoting neuroinflammation, senile plaque formation and accumulation of toxic neurofibrillary tangles [17,125]. In a healthy brain, small amounts of soluble amyloid beta peptide (Aβ) are produced and degraded through enzymatic, proteasomal and lysosomal machineries [124,126]. In brains affected by AD, however, the brain performs insufficient degradation leading to an accumulation of Aβ fragments within proteinopathic brain plaques [17,124]. Furthermore, these peptides contain toxic proteoforms that impair the brain cell degradation machineries [124]. It has been recently proposed that accumulation of Aβ peptides in the brain performs a beneficial function as an antimicrobial peptide when the brain faces pathogen infections. However, if there is an overaccumulation of Aβ peptides, either due to prolonged colonization of pathogens or due to ineffectiveness in degrading them once they are no longer needed, it can lead to destruction of nearby tissue [124,127]. It has also been demonstrated that administration in mice of *P. gingivalis*, one of the main pathogenic bacteria present in periodontitis, increases intestinal permeability and, as a consequence, facilitates the transfer of LPS through the intestinal barrier [48,128]. This leakage of LPS leads to upregulated serum LPS levels, which in turn have been demonstrated to trigger systemic inflammation [48,128,129]. Repeated oral application of *P. gingivalis* induces neurodegeneration and the formation of extracellular Aβ42 in young adult wild type mice, strongly suggesting that low grade chronic periodontal infection with this pathogen can result in the development of neuropathology consistent with that of AD [130].

Oral pathogens, such as *P. gingivalis*, have also been investigated using post-mortem human brain tissues [63,127]. ApoE−/− [105,115] and pathogen-free BALB/c mice [127], and/or various *Spirochaetes*, have been reported to colocalize with Aβ plaques [124,127,131,132]. Moreover, dysbiosis of the oral and intestinal microbiotas can potentially initiate and accelerate the formation of Aβ plaques and neurofibrillary tangles [17]. As explained above, periodontitis is a dysbiotic immunoinflammatory disease that can directly mediate neuroinflammation [133,134,135]. Several studies claim that chronic periodontal inflammation can induce changes in the gut microbiota, increasing individual inflammatory responses. This is because periodontitis can cause a secretion of bacterial pathogens, such as LPS or peptidoglycans, which have been considered as modifiable risk factors for AD [136]. As previously indicated in this work, periodontitis has been associated with increased risk of dementia through the mechanisms of systemic inflammation [117,137,138]. Another study corroborates the fact that oral microbiota may influence the risk of AD through systemic access to the brain of imbalanced oral microbiota strains and hypothesizes the relationship that AD neuropathology may establish with periodontitis through this mechanism [139]. The former study takes into account that chronic periodontitis is significantly linked to increased risk of suffering AD and other age-related dementias [139]. It was also demonstrated that AD subjects present lower diversity of microorganisms in the oral microbiota compared to healthy controls, a fact that points to a particular AD-linked dysbiosis of the oral cavity [28]. Changes in the oral microbiome (e.g., due to predominant adoption of western diet) may result, according to this study [136], in intestinal dysbiosis, which in turn leads to low-grade inflammation in the intestine and increased permeability of biological barriers, including the blood–brain barrier (BBB). Consequently, neuroinflammation and cognitive impairment may appear as subsequent factors to oral dysbiosis based on the assumptions reported in these studies. Furthermore, oral pathogens such as *P. gingivalis* lead to an ‘oralization’ of the intestinal microbiota [134,140], a mechanism that produces intestinal inflammation, and may be linked to apparition and sustainment of neuroinflammation [128,141] through translocation of oral/intestinal toxic bacterial proteases to the brain [136]. Classical inflammatory mediators, such as eicosanoids and cytokines, may also contribute to neurodegeneration as detailed. Docosahexaenoic acid (DHA) is a fatty acid that plays a fundamental role in neural function and exhibits anti-inflammatory properties by inhibiting the production of pro-inflammatory mediators, such as eicosanoids and cytokines. It has been reported that high consumption of DHA-rich fish significantly reduces the probability of developing AD [142], and that a consumption of 900 mg/day of DHA can even provide neuroprotection while early-stage dementia-related cognitive deficits appear [143]. DHA deficiency occurs due to increased lipid peroxidation mediated by free radicals, dietary intake deficit and/or impaired hepatic DHA administration to the brain [144]. DHA has been assigned with several neuroprotective abilities, such as blocking the cascade between Toll-like/cytokine receptors and NF-κB activation. Toll-like receptors (TLRs) are transmembrane receptors that initiate signals in response to different stimuli, such as tissue injury and infection. It is now recognized that the lipid components of the diet can modulate transmembrane TLRs and the consequent immune and inflammatory responses. These receptors are expressed in a variety of cell types related to immunity in mammals and are also present in microglia, astrocytes, neurons and oligodendrocytes. Recently, there is evidence linking them to neurodegenerative conditions (53). In the same vein, various components of the Mediterranean diet, such as the fatty acids of long-chain w-3 fish derivatives, and plant polyphenols, including resveratrol, have been linked with positive effects on brain health [145,146].

In a related vein, recently, Ribeiro-Vidal et al. [147] investigated the in vitro antimicrobial properties of two w-3 fatty acids, DHA and eicosapentaenoic acid (EPA). The authors used a subgingival biofilm as model in their studies. The results showed that both DHA and EPA exerted significant reduction on the harmful bacterial strains studied, such as *P. gingivalis*, *A. actinomycetemcomitans*, *F. nucleatum* and *Veillonella parvula*, among others [147]. All in all, more randomized controlled trials are needed to make better recommendations towards the acquisition of a healthy microbiota [146]. There are also multiple studies on the effect of various types of anthocyanins, which are an example of polyphenols, related to a positive impact on the prevention and amelioration of certain clinical manifestations of progressive AD. Among these, a literature review concluded that the intestinal microbiota has a significant impact on the pathogenesis of AD, which could be clinically delayed following administration of anthocyanins [148,149,150]. According to another study conducted in 2020, the neuroprotective ability of cyanidin-3-glucoside (C3G) was also reveled in a mice model of AD [151]. These studies indicate that is important to analyze the effects of these promising molecules on the oral microbiota to further understand if this type of microbiota is also implicated in the neuroprotective effects exerted by these compounds, as already demonstrated for gut microbiota.

It is known that oral status may be an important factor for general health and a diverse and well-developed microbiota has been associated with overall well-being. Thus, prevention of oral pathologies and inhibition of the protease of *P. gingivalis* or other bacteria associated with periodontitis and AD, such as *A. actinomycetemcomitans*, *Actinomycetales* and *Prevotella* [137,139], *T. forsythia*, *E. coli*, *Chlamydiapneumonia* and *F. nucleatum* may help to reduce the current neurodegenerative burden [152], an extremely compelling hypothesis that requires of further investigation. It has been presupposed that the oral microbiota remains in the oral cavity and does not have the ability to preferentially reach the intestine or other body sites. However, it has been already proven that the oral microbiota can easily reach the intestine or lungs of people who have a compromised immune system, being able to trigger inflammation and health issues, as reviewed here, at an organismic level [28,48].

### 4.4. Oral and Gut Microbiota in Chronic Human Diseases

Although the general vision that oral bacteria, under normal conditions of ingestion, are unable to colonize a healthy intestine is the most accepted, high levels of oral microbes have been found in the gut microbiota of patients with different chronic human diseases, including colon cancer, inflammatory bowel disease and liver cirrhosis [153]. The former authors indicate that *Klebsiella spp.* can migrate from the saliva and colonize the gut when this former organ presents dysbiosis [153] In fact, more than 1000 mL of saliva is produced by adults daily and the majority of that amount enters the gastrointestinal tract. Accordingly, oral microbiota, which is an important reservoir of intestinal microbes, should exert an important role in maintaining the stability of the intestinal microecosystem [153]. A clear example of the implications of both microbiota populations in human diseases is diabetes mellitus (DM), as it has been shown as Fusobacteria and Actinobacteria were more abundant in subjects with diabetes, while Proteobacteria were less abundant in the oral cavity of these subjects, indicating an orchestrated dysbiotic profile simultaneously affecting the oral and the gut microbiota [153]. However, the relationship between these two crucial organism microbiota is only incipiently uncovered and needs further efforts to be properly understood at the context of several other major diseases including cardiovascular and specifically AD.

## 5. Conclusions

This systematic review highlights the importance of the oral microbiota in health and pathological conditions and the diet’s modulatory capacity over the oral microbiota. The relationship between the oral microbiota and oral diseases has been analyzed. Indeed, according to multiple recent studies, there is clear evidence of the relationship between dysbiosis of the oral microbiota and cardiovascular and neurodegenerative diseases. The recent development of molecular genetics is of paramount importance for the study of the oral microbiota and its association with oral and general health. Likewise, microbiomics and metagenomics are two areas of research that have emerged to identify the presence of specific bacteria in the body, and thus, understand the nature of microbial activity during health and disease [43].

The high sensitivity of oral microorganisms could predict subtle changes in health status and serve as a potential biomarker for early detection of disease [69]. Moreover, it may be possible to modulate the oral microbiota even in adults through changes in the dietary pattern, being able to make recommendations for the prevention of the appearance of oral pathologies [40]. It is very necessary to continue with more research, especially at the clinical level, to continue studying how to regulate, modify and improve the oral microbiota from nutrition, in order to improve general health in people. In addition, it is worth noting the importance of being able to treat and prevent possible disorders or pathologies especially through food and food supplements, in addition to following a healthy lifestyle.

The oral microbiota presents a crucial factor, since it is in contact with the external environment. This is fundamental since this factor is modifiable, and thus, could improve the individual health of the population. Since the broad spectrum of beneficial and potentially pathogenic bacteria are present in practically all microbiomes, a crucial path would be to favor the growth of beneficial species and moderate biofilm growth and metabolism to reduce dysbiosis. That is why it is of great relevance to know the optimal balance of the microbiota and also to know when there is a dysbiosis. Moreover, it may highlight the importance of more studies combining modifications in the diet and analyzing the impact that this factor has on the microbiota and in both oral and general people’s health.

Another important aspect to consider is that a human safety assessment combined with health risk assessment is needed to better protect people from potential external hazards, whether the risks come from microbial pathogens, environmental chemicals or physical agents, such as radiation, medication or food additives. Tools are needed to identify hazards and assess exposure risks that may be problematic to individuals and populations [7]. Finally, it is not enough just to carry out an intervention in the diet and microbiota to improve the oral and general health of people, since it is understood that to avoid the disease, it is also necessary to take into consideration and improve other habits, such as increasing physical activity and reducing stress or improving the management of the latter. Maintaining a balance between all these pillars can be a good strategy to maintain a balanced microbiota, thus increasing the chances of maintaining proper systemic health.

## Figures and Tables

**Table 1:** Main factors influencing the compositions of the oral microbiota.

Age	Host and Environment	Habitat	Biofilm Maturation
Changes in the host and its habits	Genetic factors	Surface ^1^	Environment
Microevolution	Diet and lifestyle Immune system	Oxygen	Probiotics
Horizontal transfer of microorganisms	Changes in host defenses	Nutritional status	Oral hygiene
Changes in diversity	Broad spectrum antibiotics	Oral hygiene	Microbial interactions
	Hormonal balance	pH	Immune response
	Environment	Cell flaking in the mucosa	Density
		Salivary flow and gingival crevicular fluid	

^1^ Tooth, mucosa, subgingival groove, tongue.

**Table 2:** Systemic diseases and pathologies related to dysbiosis of the oral microbiome.

Autoimmune Disorders	Metabolic and Inflammatory Diseases	Cancer Diseases	Neurodegenerative Diseases
Rheumatoid arthritis [42,73]	Non-alcoholic hepatic steatosis	Colorectal cancer (*F. nucleatum*)	Multiple sclerosis
Sjögren syndrome, systemic lupus erythematosus [32]	Insulin resistance, diabetes, atherosclerosis [74]	Pancreatic cancer (*P. gingivalis* and *A. actinomycetemcomitans*)	Dementia
Inflammatory bowel disease [29]	Chronic kidney disease	Gastrointestinal cancer [32]	Alzheimer’s disease
	Hypertension, stroke, obesity	Head and neck tumors	
		Oral cancer [75]	
